# Examining parent and youth experiences of familism: Effects on youth well‐being and family dynamics

**DOI:** 10.1111/jora.70082

**Published:** 2025-09-25

**Authors:** Gianna Rea‐Sandin, Sylia Wilson

**Affiliations:** ^1^ Department of Pediatrics University of Minnesota Minneapolis Minnesota USA; ^2^ Institute for Child Development University of Minnesota Minneapolis Minnesota USA

**Keywords:** cultural values, early adolescence, familism, measurement

## Abstract

This study tested whether measurement of the Familism scale of the Mexican American Cultural Values Scale (comprising Support, Obligations, and Referent subscales) was invariant across parent and youth reporters in early adolescence and examined whether reporter discrepancies predicted youth functioning across substance use, problem behavior, academic, peer, and family domains 1 year later. The sample comprised 2410 multi‐ethnic Hispanic/Latino/a youth (*M*_age_ = 12.87 years; 48% female) and their parents from the Adolescent Brain Cognitive Development (ABCD) Study. At least partial metric invariance was established for Support and Obligations subscales, suggesting associations between mean levels of these subscales with other measures can be meaningfully compared by parent and youth reporters. However, the Referent subscale and Total Familism scale demonstrated only configural invariance, meaning their structure was similar across reporters, but item loadings, latent means, and associations with other measures were not comparable. Reporter discrepancies in Support and Obligations did not account for unique variance in any indicator of youth functioning beyond the main effects of parent and youth Support and Obligations, both of which were associated with adaptive youth outcomes. Both parent and youth reports on the Familism scale of the Mexican American Cultural Values Scale have demonstrated reliability and validity in previous work, but our tests of measurement invariance suggest only the Support and Obligations subscales, but not the Referent or Familism scales, can be meaningfully compared across parent and youth reporters. This work has important implications for the assessment of familism in early adolescence and its role for youth well‐being.

## INTRODUCTION

As youth move through adolescence, family dynamics and endorsement of family values show normative developmental shifts (Stein et al., 2014). Familism, a set of cultural values commonly endorsed in Hispanic/Latino/a families that emphasize support, respect, closeness, loyalty, and obligation to the family (Cahill et al., 2021), has been shown to be important across nearly every domain of adolescent functioning, including substance use, mental health, academic performance, peer relationships, and family functioning (Cahill et al., 2021; Hernández & Bámaca‐Colbert, 2016; Knight et al., 2010; Rea‐Sandin, Toro, & Wilson, 2023; Stein et al., 2015). There has been work establishing that measures of familism are psychometrically equivalent by racial/ethnic group, including Hispanic/Latino, European American, Black, Asian American, and Multiracial youth (Christophe & Stein, 2022; Mahrer et al., 2019; Soli et al., 2009). However, although studies have considered parent and youth reports of familism using well‐established measures (e.g., Mexican American Cultural Values Scale; Knight et al., 2010), whether the meaning of responses is equivalent across reporters has not yet been examined.

Establishing measurement invariance, including the latent construct structure, whether the structure is comparably indexed by measure scales and items, and the extent to which measure scores are comparably related to latent scores, is necessary to determine whether observed similarities or differences among reporters reflect true effects or are confounded by systematic measurement error (Agi‐Garratt et al., 2023). That is, interpretation of the meaning, function, and implications of reports of familism made by different reporters, including parents and youth, requires invariance in measurement across reporters.

In addition, the transition into adolescence is a developmental period during which parents and youth may not align on their endorsement of familism (e.g., Rea‐Sandin, Toro, & Wilson, 2023), as youth must reconcile their desires for increasing autonomy with maintaining familial values and expectations set by their parents (Stein et al., 2014). Literature suggests that discrepancies in levels of familism among parents and youth are associated with poorer youth outcomes, including poor self‐esteem and greater externalizing behaviors (Baumann et al., 2010; Toro & Nieri, 2018). However, this research has primarily relied on parent–youth difference scores, which have been shown to be poor statistical indicators of informant discrepancies (see Laird & De Los Reyes, 2013). In addition, there is also a need to examine whether reporter discrepancies are associated with other domains of adolescent functioning, including academic performance, peer relationships, and family functioning.

To evaluate the equivalence of measurement of parent–youth reports of familism, we tested whether the Familism scale of a commonly used measure of family cultural values, the Mexican American Cultural Values Scale (Knight et al., 2010), was invariant across parent and youth reports in the multi‐ethnic Hispanic/Latino/a subsample of the Adolescent Brain Cognitive Development (ABCD) Study, a large, demographically diverse sample of adolescents in the United States (Garavan et al., 2018). If measurement invariance by reporter could be established, we then examined whether discrepancies in parent–youth reports of familism longitudinally predicted a broad range of youth outcomes, including substance use, mental health, academic, peer, and family domains. Hispanic/Latino/a youth experience greater mental and physical health, as well as educational disparities compared with their non‐Hispanic/Latino/a peers. Thus, it is crucial that measures assessing the potential protective cultural factors that foster positive development for Hispanic/Latino/a youth, such as familism, can be meaningfully interpreted in the same manner when reported by both parents and youth (McLaughlin et al., 2007; Rea‐Sandin et al., 2021).

### Familism

Hispanic/Latino/a individuals are considered the largest minoritized racial/ethnic group in the United States (Jones et al., 2020), and although they represent an incredibly heterogeneous and diverse group of individuals with various national origins, migration histories, and socioeconomic resources (Umaña‐Taylor & Fine, 2001), collectively, this group has been shown to share a strong emphasis on prioritizing family‐oriented values (Calzada et al., 2014; Sabogal et al., 1987; Villarreal et al., 2005). Familism values provide a guide for how family members are expected to behave and interact with one another and have been cited as integral for Hispanic/Latino/a families in the United States for effectively navigating various physical and emotional stressors (Sabogal et al., 1987; Stein et al., 2014; Sumner et al., 2018).

Familism values include attitudes surrounding the desirability of maintaining close family relationships (Support), the importance of tangible caregiving within the family (Obligations), and the reliance on the family to define one's identity (Referent; Knight et al., 2010). Theoretical and empirical work suggests that familism values promote adaptive youth development (Bustos & Santiago, 2022; Coll et al., 2000; Hernández & Bámaca‐Colbert, 2016; Kapke et al., 2017), and there is a robust body of literature conducted in adolescence that demonstrates the positive role of both parent‐ and youth‐reported familism on youth adjustment, including decreased substance use behaviors, fewer behavioral problems, better academic performance, greater prosocial behaviors, and greater family cohesion (Cahill et al., 2021).

### Familism discrepancies among parents and youth

Much of the research on familism focuses on middle to late adolescence (Stein et al., 2014). However, previous work suggests that discrepancies between parents and youth in reports of familism are evident beginning in the late childhood/early adolescent period, with research showing that parent and youth reports of familism demonstrate little to no association across at this age (Germán et al., 2009; Rea‐Sandin, Toro, & Wilson, 2023). Early adolescence is a developmental period where youth begin to perceive and internalize family values (Cornwell et al., 1996; Grotevant, 1983). Specifically, youth are beginning to discern which values they wish to adopt in their own belief systems, which may or may not align with their parents. Examination of youth endorsement of familism in late childhood and early adolescence offers the opportunity to identify parent–youth discrepancies in familism as they begin to emerge, and as a result, examine whether discrepancies matter for youth functioning at this age (Stein et al., 2014).

Informed by theory from the acculturation literature, this study draws on the acculturation gap‐distress hypothesis, which posits that the misalignment of values between parents and children could increase conflict within the family, leading to adolescent maladjustment (Lee et al., 2000; Lui, 2015; Szapocznik et al., 1978; Telzer, 2011). Additionally, the problem suppression‐facilitation model suggests that the manifestation of socioemotional problems is partially due to the values, beliefs, and expectations that are promoted or discouraged within the family unit (Weisz, 2013). Applied to familism, these frameworks suggest that the extent to which parents and youth are aligned (or not aligned) on familism values may cause stress and disharmony in the family dynamic, in turn leading to adolescent maladjustment (Shukla et al., 2025).

Findings from work examining the implications of differences in parent–youth reports of family cultural values, including familism and related constructs like acculturation, have been mixed. For example, a study of Latina adolescents found that greater discrepancies in mother‐ and daughter‐reported familism were associated with poorer mother‐daughter mutuality (attunement among adolescents and their mothers) and greater adolescent externalizing behaviors, which, in turn, predicted Latina adolescent suicide attempts (Baumann et al., 2010). Similarly, a study of Latino immigrant families found that greater discrepancies in parent–adolescent family cultural values were associated with poorer adolescent self‐esteem and more aggressive behaviors (Toro & Nieri, 2018). Parent–adolescent differences in acculturation have also been found to be associated with greater risk for substance use in adolescence (Elder et al., 2005). However, other studies report nonsignificant associations between parent–adolescent discrepancies on family cultural values and outcomes, including externalizing behaviors and parent–youth conflict (Lau et al., 2005; Padilla et al., 2016). Less is known about whether parent–youth familism discrepancies are associated with other salient adolescent outcomes, such as academic performance or peer relationships. Leveraging extensive measurement of relevant constructs in the ABCD Study, we considered the role of parent–youth familism discrepancies across an array of adolescent outcomes: substance use, problem behaviors, academic performance, peer relationships, and family conflict.

### Approaches to modeling parent–youth discrepancies

Previous research has taken several approaches to modeling parent–child discrepancies. One method involves the creation of categorical classifications, where reporter scores are dichotomized (e.g., high vs. low) and cross‐tabulated to create matched/mismatched groups (Lau et al., 2005; Toro & Nieri, 2018). Although easy to interpret, this approach can lead to reduced statistical power and the oversimplification of variation by defining arbitrary cutoffs (Altman & Royston, 2006).

Another frequently used approach is the use of difference scores, which are calculated by subtracting the youth's score from their parent's (Baumann et al., 2010; Elder et al., 2005). Difference scores provide a direct estimate of the magnitude and direction of parent–youth differences, but have well‐documented limitations, including low reliability, collinearity with main effects, and potential loss of information about the individual contributions to the discrepancy (Edwards, 2001; Laird & De Los Reyes, 2013). To address this, some researchers have also included the mean score of each reporter as covariates in regression models, allowing for examination of whether discrepancies have an effect above and beyond mean endorsement (Padilla et al., 2016). However, this approach still assumes linear and symmetric discrepancy effects, which may not accurately reflect the true nature of informant discrepancies (Laird & De Los Reyes, 2013).

Polynomial regression has been cited as a flexible and robust alternative to traditional difference score approaches for testing informant discrepancies (Edwards, 1994; Laird & De Los Reyes, 2013). Unlike categorical approaches, this approach avoids the creation of arbitrary cutoffs by including informant reports as continuous variables and allows for testing of nonlinear effects. Polynomial regression leverages interaction and quadratic terms, in addition to main effects, to model how outcomes vary not only by each informant's endorsement but also by the degree to which there is alignment or divergence between informants. Specifically, interaction terms capture whether the effect of one reporter's endorsement depends on the level of the other, allowing for a direct test of whether reporter discrepancies are associated with the outcome of interest. Quadratic terms indicate the presence of nonlinear effects. For example, using internalizing symptoms as the outcome, a significant positive quadratic effect of youth‐reported familism suggests that both low and high endorsement are associated with higher internalizing symptoms. Polynomial regression also avoids assumptions of symmetry by allowing the direction of discrepancies to have distinct effects. For example, youth > parent discrepancies may differentially relate to youth functioning compared with parent > youth discrepancies. Thus, the analytic flexibility makes polynomial regression particularly well‐suited to examine whether and how parent–youth familism discrepancies relate to youth functioning.

### Measurement invariance by reporter

Before we can confidently assert that parent–youth discrepancies in reports of familism values reflect actual substantive differences among reporters, and not the effect of construct‐irrelevant measurement bias, it is crucial to demonstrate that the meaning of responses is equivalent across parents and youth (Maassen et al., 2023). Measurement invariance analyses provide a test of psychometric equivalence of a construct across groups or over time (see Putnick & Bornstein, 2016). Configural, metric, and scalar invariance reflect increasingly stringent models that are tested in order to establish measurement invariance, or equivalence (Agi‐Garratt et al., 2023). Configural invariance establishes that scale items and factors are equivalent for both groups (i.e., the latent constructs have the same pattern of free and fixed loadings across groups), meaning the construct structure of the measure is comparable across groups or over time. If configural invariance is established, metric invariance can then be tested to demonstrate that the strength of factor loadings is comparable across groups, meaning associations between the construct and other variables can be compared across groups. If metric invariance is supported, scalar invariance is tested, which establishes that the item intercepts are equivalent across groups, meaning that responses have similar meanings across groups.

The Mexican American Cultural Values Scale (Knight et al., 2010) is a comprehensive, 50‐item measure that was originally created to assess cultural values among Mexican American adolescents and adults. The measure assesses both Mexican American values (Familism—Support, Familism—Obligations, Familism—Referent, Respect, Religion, and Traditional Gender Roles) and Mainstream values (Material Success, Independence and Self‐Reliance, and Competition and Personal Achievement). Consistent with prior research (e.g., Mahrer et al., 2019; Padilla et al., 2016; Rea‐Sandin, Toro, & Wilson, 2023; Son et al., 2024), the present study considered the three familism subscales of the Mexican American Cultural Values Scale to assess familism values. Specifically, Familism—Support assesses emotional and instrumental support within the family, Familism—Obligations taps into perceived responsibilities family members have toward one another, and Familism—Referent includes items regarding using the family to define oneself. These subscales, when considered together, have demonstrated strong psychometric reliability (Calderón‐Tena et al., 2011; Knight et al., 2010; Wheeler et al., 2017).

To date, research has been primarily focused on testing the invariance of familism measures (including the Mexican American Cultural Values Scale) across racial/ethnic groups (Christophe & Stein, 2022; Miles et al., 2012; Schwartz, 2007; Schwartz et al., 2010), as well as within Hispanic/Latino/a subgroups (Villarreal et al., 2005). The Mexican American Cultural Values Scale also demonstrated evidence of strong (metric) longitudinal measurement invariance across late childhood/early adolescence (Knight et al., 2016). In addition, the authors of the Mexican American Cultural Values Scale found that both parent‐ and adolescent‐reported cultural values demonstrated a comparable latent structure (Knight et al., 2010), consistent with configural invariance. However, to our knowledge, formal tests of measurement invariance for the familism subscales of the Mexican American Cultural Values Scale by reporter have not yet been conducted. To interpret parent and youth reports of familism and similarities and differences across reporters as meaningful, as opposed to measurement artifacts, it is necessary to establish that familism is being equivalently measured across reporters.

### Current study

The first goal of this study was to test whether measurement invariance of the Familism scale and subscales from the Mexican American Cultural Values Scale (Knight et al., 2010) could be established by reporter (parents and youth). The second aim of the study was to assess whether discrepancies in parent and youth reports of familism in late childhood (age 11–12 years) predicted youth functioning in substance use, mental health, academic, peer, and family functioning domains 1 year later (age 12–13 years). We hypothesized that parent–youth discrepancies in reports of familism would be associated with poorer youth outcomes, including earlier substance use initiation, higher endorsement of problem behaviors, poorer academic performance, and greater family conflict. We addressed these aims using the ABCD Study, as the ABCD Study's large sample of Hispanic/Latino/a youth and extensive measurement make it an ideal dataset to address the goals of the current study.

## MATERIALS AND METHODS

### Participants and procedures

Participants were drawn from the longitudinal Adolescent Brain Cognitive Development (ABCD) Study (www.abcdstudy.org), which includes 11,875 youth and their parents/caregivers who were recruited from 21 sites across the United States. Parents provided consent, and youth provided assent at each wave of the study. Data for this study were drawn from the ABCD 5.1 data release (DOI: https://doi.org/10.15154/z563‐zd24), which includes full data from the 2‐year follow‐up (*n* = 10,972, youth age 11–12) and the 3‐year follow‐up (*n* = 10,335, youth age 12–13), accessed using the ABCD NIH data archive in July 2024. We included youth and their parents who endorsed being Hispanic/Latino/a (1 = yes on demo_ethn_v2 variable of abcd_p_demo datafile). The analytic sample comprised *n* = 2410 youth (48.03% female). This study was preregistered. There were only two minor deviations from the preregistration, and they are noted in the Measures and Analytic Plan.

### Measures

#### Familism

Parents and youth each completed the Familism scale (16 items; see Table S1 for items) of the Mexican American Cultural Values Scale (Knight et al., 2010) at youth age 11–12 years. The Familism scale includes 16 items assessing familism values that are rated on a 5‐point Likert scale ranging from 1 (‘not at all’) to 5 (‘completely’). The Familism scale comprised three subscales: Support (six items; e.g., ‘Family provides a sense of security because they will always be there for you’), Obligations (five items; e.g., ‘Children should be taught that it is their duty to care for their parents when their parents get old’), and Referent (five items; e.g., ‘Children should always do things to make their parents happy’). Sum scores of items within subscales were used (Support: *α*_parent_ = .83, *α*_youth_ = .80; Obligations: *α*_parent_ = .72, *α*_youth_ = .77; Referent: *α*_parent_ = .79, *α*_youth_ = .84 at age 11–12), as well as Total Familism (*α*_parent_ = .91, *α*_youth_ = .92 at age 11–12). Note that the ABCD Study administered a set of the subscales from the Mexican American Cultural Values Scale (i.e., Familism—Support, Familism—Obligations, Familism—Referent, Religion, and Independence/Self‐Reliance). We focused on the Familism subscales to maintain comparability with previous research and to ensure conceptual consistency in the measurement of familism values.

#### Substance use

Youth completed the Lifetime Use Interview (Lisdahl & Price, 2012; Sullivan et al., 2022), which assessed whether they had used any of the following substances/drugs: alcohol, nicotine products, cannabis products, synthetic cannabinoids, cocaine, cathinones, methamphetamine, ecstasy/MDMA, ketamine, gamma‐hydroxybutyrate (GHB), heroin, psilocybin, salvia, other hallucinogens, anabolic steroids, inhalants, and prescription misuse of stimulants, sedatives, opioid pain relievers, and over‐the‐counter cough/cold medicine. Substance use initiation was indexed as a sum score of all substances that were endorsed as used across each wave from age 9–10 years to age 12–13 years.

#### Mental health

##### Parent‐reported symptoms

Parents completed the Child Behavior Checklist (CBCL; Achenbach, 2009) at youth age 12–13. The CBCL includes 112 items assessing depression, anxiety, somatic, rule‐breaking, aggression, and other problems over the past 6 months that are rated on a 3‐point Likert scale ranging from 0 (‘Not True (as far as you know)’), 1 (‘Somewhat or Sometimes True’), or 2 (‘Very True or Often True’). The raw sum score of total problems was used (*α* = .95 at age 12–13).

##### Youth‐reported symptoms

Youth completed Brief Problem Monitor (BPM; Achenbach, 2009) at youth age 12–13. The BPM includes 19 items from the parent‐reported CBCL assessing depression, anxiety, somatic, rule‐breaking, aggression, and other problems symptoms over the past week that are rated on a 3‐point Likert scale ranging from 0 (‘Not True (as far as you know)’), 1 (‘Somewhat or Sometimes True’), or 2 (‘Very True or Often True’). The raw sum score of total problems was used (*α* = .83 at age 12–13).

#### Academic functioning

##### School connectedness

Youth completed the School Risk and Protective Factors (SRPF; Zucker et al., 2018) at youth age 12–13. The SRPF includes 12 items assessing school connectedness that are rated on a 4‐point scale ranging from 1 (‘Definitely true’), 2 (‘Mostly true’), 3 (‘Mostly not true’), and 4 (‘Definitely not true’). The SRPF includes three subscales: school climate/environment (six items; e.g., ‘I get along with my teachers’), personal involvement in school (four items; e.g., ‘In general, I like school a lot’), disengagement from academic goals (two items, reverse scored; e.g., ‘Usually, school bores me’). A sum score of all items was used (*α* = .81 at age 12–13).

##### Academic performance

The original study preregistration only included school connectedness as a measure of academic functioning. To obtain a more well‐rounded assessment of youth's academic environment, we opted to additionally include a measure of parent‐reported grades. Parents reported on the question ‘What grades did your child receive in school last year?’ beginning at youth age 11–12, and we considered reports at youth age 12–13. Response options ranged from 1 to 12, with higher scores indicating poorer academic performance. This variable was recoded so that higher scores indicated better academic performance, with scores ranging from 1 (‘97–100/4.0 or higher/Exceeding the standards’) to 12 (‘0–65/0.00–0.99/Does not meet minimum standards’).

#### Peer influences

##### Peer affiliation

Youth completed the Youth Peer Behavior Profile (YPBP; Bingham et al., 1995) at youth age 12–13. The YPBP includes six items assessing how many of their friends have engaged with prosocial and rule‐breaking behaviors on a scale ranging from 1 (‘None or almost none’), 2 (‘A few’), 3 (‘Half’), 4 (‘Most’), and 5 (‘All or almost all’). Prosocial peer behaviors include three items (i.e., are athletes, go to church once a month or more, or are excellent students) and rule‐breaking peer behaviors include three items (i.e., have skipped school, have been suspended from school, or have shoplifted occasionally). We considered prosocial and rule‐breaking peer affiliation as separate outcomes, given their weak associations (*r* = .03, *p* = .25 at age 12–13).

##### Peer victimization

Youth completed the Revised Peer Experiences Questionnaire (PEQ; Barch et al., 2021; Prinstein et al., 2001) at youth age 12–13. The PEQ includes nine items assessing aggressive behavior toward peers on a 5‐point scale indicating how often the adolescent engaged in the behavior: 1 (‘Never’), 2 (‘Once or twice’), 3 (‘A few times’), 4 (‘About once a week’), and 5 (‘A few times a week’). The measure assessed whether the adolescent engaged in overt (mentally or physically harming others, ‘I threatened to hurt or beat up another kid’), relational (using social relations to harm others, ‘I did not invite a kid to a party or other social event even though I knew the kid wanted to go’), or reputational (intentionally manipulating or damaging the social standing of a peer, ‘I tried to damage another kid's social reputation by spreading rumors about them’) aggression toward peers (Halbreich et al., 2023). A sum score was computed to represent total aggressive behaviors toward peers (*α* = .73 at age 12–13).

#### Family functioning

Parents completed the Family Conflict subscale of the Family Environment Scale (FES; Moos, 1994; Moos & Moos, 2013) at youth age 12–13. The FES includes nine items assessing their family's functioning on a binary scale (true/false), and sum scores were created, such that higher scores indicated greater familial conflict. Example items include ‘We fight a lot in our family’ and ‘Family members often criticize each other’. Reliability was fair (*α* = .68 at age 12–13).

#### Covariates

Covariates included youth sex assigned at birth assessed at baseline (1 = male), youth age at the 3‐year follow‐up wave, household income assessed at age 9–10, and parental education assessed at age 9–10.

##### Family income

At baseline, parents reported on their total household income in the past 12 months before taxes, with response options, including (1) less than $5000; (2) $5,000–$11,999; (3) $12,000–$15,999; (4) $16,000–$24,999; (5) $25,000–$34,999; (6) $35,000–$49,999; (7) $50,000–$74,999; (8) $75,000–$99,999; (9) $100,000–$199,999; and (10) $200,000 and greater.

##### Parental education

Parents reported on their highest level of education. Response options ranged from 0 (‘Never attended’) to 21 (‘Doctoral degree’). Responses were recoded as follows: 1: ‘less than 8th grade’; 2: ‘some high school’; 3: ‘high school or equivalent’; 4: ‘some college’; 5: ‘associate's degree’; 6: ‘bachelor's degree’; 7: ‘master's degree’; and 8: ‘doctoral or professional degree’. The parent with the highest education was selected and used to represent parental education.

### Missing data

Among the 2410 total Hispanic/Latino/a participants with data at the 3‐year follow‐up (age 12–13) and 11 key constructs (parent‐reported Familism at youth age 11–12, youth‐reported Familism at youth age 11–12, substance use initiation, parent‐reported problem behaviors, youth‐reported problem behaviors, school connectedness, academic performance, prosocial peer affiliation, rule‐breaking peer affiliation, peer victimization, and family conflict), *n* = 1143 (47.42%) had complete data. Youth with more complete data across the key constructs were more likely to have higher household income and parents with higher education. Youth sex was not significantly associated with data completeness across the 11 key constructs. We considered data to be missing at random (MAR), and therefore, we used full information maximum likelihood (FIML) to retain all participants.

### Analytic plan

Bivariate correlations and measurement invariance analyses were conducted using MPlus 7.0 (Muthén & Muthén, 2017), with the *type = complex* command that accounts for the nonindependence of individual‐level data (Hatoum et al., 2018). In addition, the *cluster* command was used to account for nesting within families, and the *stratification* command was used to account for clustering within the study site. FIML with robust standard errors (MLR) was used to include all possible participants and produce unbiased parameter estimates and standard errors under MAR and missing completely at random (MCAR) (Enders & Bandalos, 2001; Muthén & Kaplan, 1985). Polynomial regression analyses were conducted in R using the lme4 package (Bates et al., 2015), incorporating random effects to account for clustering by family and study site.

#### Measurement invariance

We conducted measurement invariance testing to examine whether the Support, Obligations, and Referent subscales, as well as Total Familism, showed measurement invariance by reporter (parent and youth). For each test, we compared the following models: (1) freely estimating factor loadings (configural invariance), (2) constraining factor loadings to be equal by reporter (metric, or weak invariance), and (3) constraining factor loadings and intercepts to be equal by reporter (scalar, or strong invariance).

When establishing the baseline measurement models, we included the ‘MODINDICES’ command in Mplus. The use of this command was not included in our preregistration, but we opted to do so to identify potential model adjustments to improve model fit. For subsequent tests of metric and scalar invariance, we used the MODEL = CONFIGURAL METRIC SCALAR command in Mplus. Given that modification indices cannot be generated under this specification, we evaluated partial metric and scalar invariance by systematically freeing individual loadings or intercepts for each subscale as needed.

Models were compared using −2‐log likelihood chi‐square test of fit, as well as Root Mean Square Error of Approximation (RMSEA) < .06, Comparative Fit Index (CFI) > .95, and the Tucker Lewis Index (TLI) > .95, following conventional guidelines (Hu & Bentler, 1999). If the more constrained model does not have worse model fit compared with less constrained models (change in CFI < .010; Cheung & Rensvold, 2002), that suggests that the structure of the latent constructs, factor loadings, or intercepts, respectively, is comparable across parent and youth reporters.

#### Polynomial regression analyses

We used polynomial regression analyses to examine whether discrepancies between parent‐ and youth‐reported Familism predicted youth outcomes. Polynomial regression analyses are an improvement over traditional difference score approaches, allowing researchers to test whether interactions between parent and youth reporters provide additional information over and above the main effects of individual reporters (Nelemans et al., 2016). In other words, this approach uses interaction terms to directly test whether high (or low) scores from one reporter is more or less strongly related to the outcome when scores from the other reporter are also high (or low) (Laird & De Los Reyes, 2013). Specifically, the polynomial regression model we used is as follows: $Y=b_{0}+b_{1}P+b_{2}A+b_{3}P^{2}+b_{4}\mathrm{AP}+b_{5}A^{2}+e$, in addition to covariates. Terms b1 and b2 capture the unique linear effects of parent‐ and youth‐reported Familism at mean levels of the other reporter. Terms b3 and b5 test for quadratic associations, as the effects of parent‐ and/or youth‐reported Familism on youth functioning may be nonlinear (East & Weisner, 2009). Finally, b4 is the interaction term between parent and youth reporters, directly testing whether the association between one informant's report and the outcome depends on the level of the other. A significant b4 coefficient tells us whether discrepancies between reporters are related to youth functioning (Laird & De Los Reyes, 2013).

For continuous outcomes, we estimated linear polynomial regression models. However, for substance use initiation (a count variable of the number of substances used), we used negative binomial regression, which is a generalized version of the same approach but more appropriate for count data. Note that binomial regression models did not include quadratic terms given interpretive challenges regarding nonlinear effects with count data. All independent variables and covariates (except sex) were mean‐centered prior to analyses to increase the interpretability of regression estimates. All regression models were tested using a Bonferroni adjusted alpha level of .006 per test (.050/9).

### Transparency and openness

Study materials and code used in this analysis are publicly available at the Open Science Framework and can be accessed at https://osf.io/tz9nu/. Analyses were conducted using Mplus 7.0 (Muthén & Muthén, 2017) and R 4.4.2 (R Core Team, 2016).

## RESULTS

### Descriptive statistics and preliminary analyses

Descriptive statistics are presented in Table 1. Substance use initiation (number of substances tried), parent‐reported problem behaviors, rule‐breaking peer affiliation, and peer victimization were highly positively skewed and kurtotic. Scores from highly skewed and kurtotic variables that were ±3 SDs from the mean were winsorized to 3 SD from the mean: parent‐reported problem behaviors: *n* = 33 (1.36%), rule‐breaking peer affiliation: *n* = 33 (1.36%), and peer victimization: *n* = 50 (2.07%). Substance use initiation was a count variable and thus was not winsorized. Mean levels of parent‐reported Obligations, Referent, and Total Familism were significantly lower than mean levels of youth reports, whereas parents reported significantly higher levels of Support than youth.

**TABLE 1:** Descriptive statistics.

	*n*	Mean (SD)	Min/max	Skew/kurtosis	*p*
Support (parent‐reported)	2121	4.25 (0.61)	1/5	−0.90/1.32	.011
Support (youth‐reported)	2125	4.21 (0.62)	1.5/5	−0.79/0.56	
Obligations (parent‐reported)	2121	3.74 (0.70)	1/5	−0.26/−0.08	<.001
Obligations (youth‐reported)	2155	4.00 (0.67)	1.2/5	−0.46/0.01	
Referent (parent‐reported)	2121	3.55 (0.78)	1/5	−0.39/0.01	<.001
Referent (youth‐reported)	2154	3.90 (0.74)	1/5	−0.45/−0.17	
Total Familism (parent‐reported)	2133	3.78 (0.64)	1/5	−0.54/0.68	<.001
Total Familism (youth‐reported)	2133	4.05 (0.62)	1.5/5	−0.54/0.14	
Substance use initiation	2410	0.20 (0.44)	0/5	2.66/13.34	
Problem behaviors^a^ (parent‐reported)	1491	16.17 (16.93)	0/107	1.83/7.00	
Problem behaviors (youth‐reported)	1790	8.32 (5.99)	0/30	0.70/0.07	
Academic performance (parent‐reported)	1944	8.81 (2.50)	1/12	−0.74/−0.07	
School connectedness (youth‐reported)	2069	37.13 (5.43)	17/48	−0.38/−0.08	
Prosocial peer affiliation (youth‐reported)	1943	8.30 (2.73)	2/15	0.08/−0.52	
Rule‐break peer affiliation^a^ (youth‐reported)	1895	3.73 (1.47)	2/13	2.26/6.74	
Peer victimization^a^ (youth‐reported)	2068	10.01 (1.84)	9/26	3.17/14.91	
Family conflict (youth‐reported)	2014	2.13 (1.84)	0/9	0.91/0.46	

*Note*: *N* = 2410. *P*‐values represent statistical significance of *t*‐tests comparing mean levels of Familism by reporter. In this study, parent‐ and youth‐reported familism was assessed at the 2‐year follow‐up (youth age 11–12), whereas parent‐ and youth‐reported problem behaviors, grades, school connectedness, prosocial and rule‐breaking peer affiliation, aggressive peer behaviors, and family conflict were assessed at the 3‐year follow‐up (youth age 12–13).

Abbreviation: SD, standard deviation.

^a^ Variables that were highly positively skewed and kurtotic were winsorized prior to analyses.

Parent‐ and youth‐reported Familism at youth age 11–12 was weakly positively correlated (Table 2). Raw bivariate correlations between parent‐ and youth‐reported Familism with the outcome variables are presented in Table S2. Parent‐reported Familism at youth age 11–12 was significantly negatively correlated with substance use initiation, aggressive peer behaviors, and family conflict, and positively correlated with school connectedness at youth age 12–13. Youth‐reported Familism at youth age 11–12 was significantly negatively correlated with substance use initiation, parent‐ and youth‐reported symptoms, aggressive peer behaviors, and family conflict, and positively correlated with school connectedness and prosocial peer affiliation at youth age 12–13.

**TABLE 2:** Bivariate correlations between parent‐ and youth‐reported familism.

	Youth support	Youth obligations	Youth referent	Youth total familism
Parent support	.10***	.10***	.11***	.11***
Parent obligations	.09***	.12***	.12***	.12***
Parent referent	.10***	.15***	.16***	.15***
Parent total familism	.11***	.14***	.14***	.14***

*** *p* < .001.

### Measurement invariance

We first fit measurement models with freely estimated factor loadings for Support, Obligations, Referent, and Total Familism separately for parents and youth to test for configural invariance. Model fit statistics for measurement models are presented in Table 3. Standardized factor loadings for final measurement models of Familism by Support, Obligations, and Referent subscales are presented in Table S3, and standardized factor loadings for the final measurement model for Total Familism are presented in Table S4. Based on recommended modification indices, the following correlated residual variance terms were included in both parent and youth models: Support: Q2 with Q12; Obligations: Q8 with Q13 and Q17 with Q22; Referent: Q9 with Q23; Total Familism: Q2 with Q12, Q8 with Q13, Q3 with Q4, and Q18 with Q27. Final model fit was excellent for Support, Obligations, Referent, and Total Familism for both reporters (see Table 3), though models for youth‐report consistently had better fit statistics (i.e., lower RMSEA and higher CFI/TLI). Thus, configural invariance was established for Support, Obligations, Referent, and Total Familism across reporters. We next fit measurement models with factor loadings constrained to be equal across parents and youth to test for metric invariance and with factor intercepts additionally constrained to be equal to test for scalar invariance. Model fit statistics are presented in Table 4.

**TABLE 3:** Model fit statistics for measurement models of familism.

Subscale	Model	Reporter	χ^2^	df	*p*	RMSEA	CFI	TLI
Support	Initial	Parent	128.15	9	<.001	.079	.940	.900
		Youth	25.366	9	.003	.029	.993	.988
	Final	Parent	60.485	8	<.001	.056	.974	.950
		Youth	24.277	8	.002	.031	.993	.987
Obligations	Initial	Parent	235.65	5	<.001	.147	.826	.652
		Youth	42.136	5	<.001	.059	.978	.955
	Final	Parent	23.39	3	<.001	.057	.985	.949
		Youth	9.697	3	.021	.032	.996	.987
Referent	Initial	Parent	149.097	5	<.001	.116	.928	.855
		Youth	44.602	5	<.001	.061	.983	.966
	Final	Parent	73.998	4	<.001	.091	.965	.912
		Youth	27.235	4	<.001	.052	.990	.975
Total	Initial	Parent	1949.209	104	<.001	.091	.791	.759
		Youth	883.099	104	<.001	.059	.925	.913
	Final	Parent	1133.560	100	<.001	.070	.883	.860
		Youth	616.952	100	<.001	.049	.950	.940

*Note*: Support, Obligations, Referent, and total models were run separately. The final models reflect the inclusion of correlated residual variance terms: support: Q2 with Q12; Obligations: Q8 with Q13 and Q17 with Q22; Referent: Q9 with Q23; total: Q2 with Q12, Q8 with Q13, Q3 with Q4, and Q18 with Q27. Final models were used for measurement invariance testing (see Table 4).

Abbreviations: CFI, Comparative Fit Index; df, degrees of freedom; RMSEA, Root Mean Square Error of Approximation; TLI, Tucker Lewis Index; χ^2^, chi‐squared.

**TABLE 4:** Measurement invariance testing for familism subscales by reporter.

Subscale	Model	Test of overall fit			Model fit relative to prior model			Fit indices			
		χ^2^	df	*p*	Δχ^2^	Δdf	*p*	RMSEA	CFI	ΔCFI	TLI
Support	Configural	86.07	16	<.001				.045	.984		.970
	Metric	94.37	21	<.001	5.031	5	.412	.040	.983	.001	.976
	Scalar	309.29	26	<.001	306.64	5	<.001	.071	.934	.050	.924
Obligations	Configural	33.60	6	<.001				.046	.991		.969
	Partial Metric	38.84	8	<.001	5.24	2	.073	.042	.990	.001	.974
	Metric	58.80	10	<.001	25.43	4	<.001	.048	.984	.007	.967
	Scalar	327.81	26	<.001	303.49	4	<.001	.102	.894	.096	.849
Referent	Configural	96.92	8	<.001				.072	.979		.949
	Metric	173.19	12	<.001	78.88	4	<.001	.079	.963	.016	.938
	Scalar	756.69	16	<.001	711.05	4	<.001	.147	.829	.150	.786
Total	Configural	1755.09	200	<.001				.060	.919		.902
	Metric	1821.19	215	<.001	52.20	15	<.001	.059	.916	.003	.906
	Scalar	3140.39	230	<.001	1716.78	15	<.001	.077	.848	.131	.841
Total—No Referent	Configural	569.89	80	<.001				.054	.953		.936
	Metric	589.57	90	<.001	19.68	10	.032	.051	.952	.001	.942
	Scalar	1458.09	100	<.001	888.20	10	<.001	.080	.871	.082	.858

*Note*: Measurement invariance testing was conducted using final models reflected in Table S1. Partial metric invariance was established for Obligations by freeing Q17 and Q22. Total – No Referent included support and Obligations subscales.

Abbreviations: CFI, Comparative Fit Index; df, degrees of freedom; RMSEA, Root Mean Square Error of Approximation; TLI, Tucker Lewis Index; ΔCFI, change in Comparative Fit Index; Δdf, change in degrees of freedom; Δχ^2^, change in chi‐squared; χ^2^, chi‐squared.

#### Support

Constraining factor loadings for the Support subscale did not result in a significant decrement in model fit relative to the configural model, establishing full metric invariance. In contrast, constraining factor intercepts led to a significant decrement in model fit relative to the metric model, and attempts to free individual item intercepts did not improve model fit, suggesting that neither full nor partial scalar invariance could be established.

#### Obligations

Constraining factor loadings for the Obligations subscale resulted in a significant decrement in model fit relative to the configural model, meaning full metric invariance could not be established. However, after freeing factor loadings for Q17 (‘Older kids should take care of and be role models for their younger brothers and sisters’) and Q22 (‘Parents should be willing to make great sacrifices to make sure their children have a better life’), the model no longer showed a significant decrement in model fit, establishing partial measurement invariance. However, constraining factor intercepts resulted in a significant decrement in model fit relative to the metric model, and attempts to free individual item intercepts did not improve model fit, indicating that neither full nor partial scalar invariance could be established.

#### Referent

Constraining factor loadings for the Referent subscale resulted in a significant decrement in model fit relative to the configural model, and attempts to free individual loadings did not improve fit. Thus, neither full nor partial metric invariance could be established, and scalar invariance was not tested.

#### Total familism

We also examined the Total Familism scale with and without the Referent subscale (i.e., mean across the Support and Obligations subscales), given that this subscale did not demonstrate even metric invariance, but neither full nor partial metric invariance could be established for either model.

Together, full metric invariance was established for the Support subscale, and partial metric invariance was established for the Obligations subscale, but only configural invariance was established for the Referent subscale and Total Familism scale.

### Polynomial regression analyses

Because measurement invariance was established only for the Support and Obligations subscales, we conducted polynomial regression analyses for those subscales. Polynomial regression analyses were conducted to examine whether discrepancies in parent and youth endorsement on the Support and Obligations subscales at youth age 11–12 predicted youth functioning 1 year later. Results for the Support and Obligations subscales (without items Q17 and Q22, which could not be constrained to be equal in metric invariance models) are reported in Tables 5 and 6, respectively. Results for the Obligations (with items Q17 and Q22) and Referent subscales, and the Total Familism scale are reported in Tables S5–S7. Results from polynomial regression analyses for the Referent subscale and Total Familism scale are reported to facilitate comparisons with previous literature, although we caution that, based on measurement invariance testing, we cannot meaningfully compare across parent and youth reporters.

**TABLE 5:** Parent and youth reports of support as predictors of youth functioning.

Model	Substance use initiation	Problem behaviors (youth‐reported)	Problem behaviors (parent‐reported)	School connectedness	Academic performance	Peer affiliation—Prosocial
Parameter	*β* (SE)	*β* (SE)	*β* (SE)	*β* (SE)	*β* (SE)	*β* (SE)
Intercept	−1.527 (.20)***	−0.473 (.08)***	0.190 (.09)*	−0.003 (.08)	−0.491 (.08)***	0.146 (.08)
Age	0.115 (.06)*	0.022 (.02)	−0.037 (.03)	−0.067 (.02)**	−0.018 (.02)	0.044 (.02)
Sex	−0.196 (.11)	0.335 (.05)***	−0.134 (.05)*	0.016 (.05)	0.347 (.05)***	−0.079 (.04)
Household income	0.107 (.07)	−0.062 (.03)	−0.060 (.04)	0.051 (.03)	0.112 (.03)***	0.151 (.03)***
Parent education	0.143 (.08)	0.050 (.04)	0.061 (.04)	−0.018 (.03)	0.136 (.03)***	0.070 (.03)*
Parent support	−0.128 (.06)*	−0.017 (.03)	−0.065 (.03)	0.027 (.03)	0.042 (.03)	0.031 (.03)
Youth support	−0.033 (.06)	−0.149 (.03)***	−0.066 (.03)*	0.212 (.03)***	−0.020 (.03)	0.144 (.03)***
Parent support^2^	–	0.015 (.02)	−0.020 (.02)	−0.008 (.02)	0.006 (.02)	−0.021 (.02)
Youth support^2^	–	−0.413 (.02)*	0.037 (.02)	0.018 (.02)	−0.028 (.02)	0.019 (.02)
Par. × Youth support	0.002 (.05)	0.011 (.02)	0.011 (.03)	0.009 (.02)	−0.003 (.02)	0.022 (.03)

Model	Peer affiliation —Rule‐break	Peer victimization	Family conflict
Parameter	*β* (SE)	*β* (SE)	*β* (SE)
Intercept	0.081 (.08)	0.166 (.08)*	0.136 (.07)
Age	0.116 (.02)***	0.037 (.02)	0.003 (.02)
Sex	−0.076 (.05)	−0.109 (.05)*	−0.043 (.04)
Household income	−0.109 (.03)***	−0.033 (.03)	0.009 (.03)
Parent education	−0.051 (.03)	0.061 (.03)	0.060 (.03)
Parent support	0.002 (.03)	−0.048 (.03)	−0.071 (.03)*
Youth support	−0.013 (.03)	−0.097 (.03)***	−0.037 (.02)
Parent support^2^	0.001 (.02)	−0.016 (.02)	−0.004 (.01)
Youth support^2^	0.035 (.02)	−0.019 (.02)	−0.017 (.01)
Par. × Youth support	−0.014 (.02)	0.022 (.02)	0.001 (.02)

*Note*: Estimates are standardized. Substance use initiation was a binary outcome (1 = yes), thus quadratic terms were not included in these regression models.

Abbreviation: SE, standard error.

* *p* < .050.

** *p* < .010.

*** *p* < .001.

**TABLE 6:** Parent and youth reports of obligations as predictors of youth functioning.

Model	Substance use initiation	Problem behaviors (youth‐reported)	Problem behaviors (parent‐reported)	School connectedness	Academic performance	Peer affiliation—Prosocial
Parameter	*β* (SE)	*β* (SE)	*β* (SE)	*β* (SE)	*β* (SE)	*β* (SE)
Intercept	−1.515 (.20)***	−0.496 (.08)***	0.146 (.09)***	−0.025 (.08)	−0.448 (.08)***	0.167 (.08)***
Age	0.109 (.06)*	0.008 (.02)	−0.030 (.03)	−0.071 (.02)**	−0.025 (.02)	0.036 (.02)
Sex	−0.210 (.11)	0.320 (.05)***	−0.140 (.05)**	0.033 (.05)	0.344 (.05)***	−0.073 (.05)
Household income	0.112 (.08)	−0.071 (.03)*	−0.049 (.04)	0.058 (.03)	0.107 (.03)***	0.159 (.03)***
Parent education	0.146 (.08)	−0.054 (.04)	0.062 (.04)	−0.015 (.03)	0.130 (.03)***	0.071 (.03)*
Parent obligations	−0.039 (.06)	−0.030 (.03)	−0.008 (.03)	0.023 (.02)	0.019 (.02)	0.060 (.03)*
Youth obligations	−0.071 (.06)	−0.113 (.03)***	−0.029 (.03)	0.165 (.03)***	−0.065 (.03)**	0.093 (.03)***
Parent obligations^2^	–	−0.009 (.02)	0.028 (.02)	−0.003 (.02)	−0.008 (.02)	−0.022 (.02)
Youth obligations^2^	–	0.024 (.02)	0.049 (.02)*	0.008 (.02)	−0.052 (.02)**	−0.010 (.02)
Par. × Youth obligations	−0.062 (.06)	0.028 (.03)	−0.027 (.03)	−0.001 (.02)	−0.002 (.02)	−0.022 (.02)

Model	Peer affiliation—Rule‐break	Peer victimization	Family conflict
Parameter	*β* (*SE*)	*β* (*SE*)	*β* (*SE*)
Intercept	0.072 (.09)	0.166 (.09)	0.113 (.08)
Age	0.121 (.02)***	0.041 (.02)	0.006 (.02)
Sex	−0.080 (.05)	−0.121 (.05)**	−0.048 (.04)
Household income	−0.114 (.03)***	−0.020 (.03)	0.016 (.03)
Parent education	−0.054 (.03)	0.051 (.03)	0.061 (.03)
Parent obligations	0.002 (.03)	−0.013 (.02)	−0.012 (.03)
Youth obligations	−0.029 (.03)	−0.064 (.03)*	−0.017 (.02)
Parent obligations^2^	−0.010 (.03)	−0.004 (.02)	0.025 (.02)
Youth obligations^2^	0.096 (.03)**	0.004 (.02)	−0.002 (.01)
Par. × Youth obligations	0.014 (.04)	−0.013 (.02)	0.009 (.02)

*Note*: These models reflect the Obligations subscale without items Q17 and Q22. Substance use initiation was a binary outcome (1 = yes); thus, quadratic terms were not included in these regression models.

Abbreviation: SE, standard error.

* *p* < .050.

** *p* < .010.

*** *p* < .001.

#### Support

The Parent‐reported Support subscale at age 11–12 predicted fewer youth‐reported numbers of substances tried and lower youth‐reported family conflict 1 year later (Table 5). The youth‐reported Support subscale at age 11–12 predicted fewer parent‐ and youth‐reported problem behaviors, greater youth‐reported school connectedness, greater prosocial peer affiliation, and lower youth‐reported peer victimization behaviors at youth age 12–13. The youth‐reported Support subscale had an accelerating, negative curvilinear association with youth‐reported problem behaviors. There were no significant interactions between parent and youth reports of the Support subscale at age 11–12 predicting youth functioning at age 12–13, suggesting no associations between parent–youth discrepancies in reports of the Support subscale and youth functioning. That is, although both the parent‐ and youth‐reported Support subscales were associated with important aspects of youth functioning, discrepancies across parent and youth reports of the Support subscale were not.

#### Obligations—Revised

The parent‐reported Obligations subscale at age 11–12 predicted greater youth‐reported prosocial peer affiliation at age 12–13 (Table 6). The youth‐reported Obligations subscale at age 11–12 predicted fewer youth‐reported problem behaviors, greater youth‐reported school connectedness and prosocial peer affiliation, and lower academic performance and youth‐reported peer victimization behaviors at age 12–13. The youth‐reported Obligations subscale had an accelerating, positive curvilinear association with parent‐reported problem behaviors and rule‐breaking peer affiliation, and the youth‐reported Obligations subscale had an accelerating, negative curvilinear association with parent‐reported academic performance. There were no significant interactions between parent and youth reports of the Obligations subscale at age 11–12 predicting youth functioning at age 12–13, suggesting no associations between parent–youth discrepancies in reports of the Obligations subscale and youth functioning.

### Sensitivity analyses

To address potential bias from missing data, polynomial regression models for the Support and Obligations—Revised subscales were re‐run using multiple imputation with *m* = 20 datasets and a fixed random seed (seed = 123). Multiple imputation results were nearly identical to those from the original models, suggesting that missing data did not substantially influence estimates. Full multiple imputation results can be found in Tables S8 and S9.

## DISCUSSION

Familism values have important implications for adolescent development and well‐being (Cahill et al., 2021). This study, which considered the Hispanic/Latino/a subsample of the ABCD Study, first tested whether the Familism scale of the Mexican American Cultural Values Scale (Knight et al., 2010) demonstrated measurement invariance by reporter (parent and youth), and then examined whether parent–youth Familism reporter discrepancies at age 11–12 predicted adolescent functioning and well‐being at age 12–13. Measurement invariance tests indicated that the Support subscale met metric invariance, and the Obligations subscale demonstrated partial metric invariance, providing evidence that the strength of the association between items and the latent factor is similar across groups (i.e., associations between these subscales with other measures can be compared by parent and youth reporter). However, the Referent subscale and Total Familism (both with and without the Referent subscale) met only configural invariance, indicating that the items load on the same factors across reporters, but the meaning or scale of the responses may differ between parents and youth. Follow‐up analyses indicated that both parent and youth reports of the Support and Obligations subscales at youth age 11–12 predicted youth well‐being 1 year later, although informant discrepancies did not account for unique variance in youth functioning over and above the individual main effect contributions of parent‐ and youth‐reported Support and Obligations subscales. Together, findings from this study have important implications for how to appropriately assess cultural values among family members and how to interpret findings across reporters.

### Measurement invariance of familism from the Mexican American cultural values scale

Measurement invariance testing demonstrated that metric invariance could be established for the Support subscale, suggesting that items in the Support subscale comparably load onto the latent factor across both parent and youth reporters. Additionally, after freeing two of the five items, the Obligations subscale met partial metric invariance, suggesting that three of the items similarly contribute to latent familism Obligations across parents and youth reporters. The two items that were freed were, ‘Older kids should take care of and be role models for their younger brothers and sisters’ and ‘Parents should be willing to make great sacrifices to make sure their children have a better life’. This suggests that parents and youth differentially conceptualize obligations within the family. For example, it is possible that parents view taking care of younger siblings as a duty or responsibility for their children, whereas youth could interpret this value as a burden. Regardless, these findings demonstrate that mean levels of the Support and revised Obligations subscales can be meaningfully compared across Hispanic/Latino/a parent and youth reporters.

However, for the Referent subscale, as well as for Total Familism, only configural invariance could be established, failing to meet more stringent tests of measurement invariance. This finding suggests that items in the Referent subscale, as well as Total Familism, do not reflect the same construct and structure across groups. Our findings are in contrast to previous work that showed comparable model fit across mother, father, and adolescent reports of the Mexican American Cultural Values Scale (Knight et al., 2010), although this study considered the entire measure, rather than individual subscales.

It is possible that items from the Referents subscale are being differentially interpreted by parents and youth. Being referent toward the family involves valuing and prioritizing family opinions; thus, interpretations of items in this subscale could depend on one's role in the family (Stein et al., 2014). For example, for the item ‘It is important to work hard and do one's best because this work reflects on the family,’ parents may interpret this in terms of professional or household contributions, whereas youth might consider their academic performance. In another example, the item ‘A person should always think about their family when making important decisions’ may be more salient for parents, as youth are still developing their autonomy and may not have had opportunities to make big decisions in their lives at this point. Together, one's role in the family and developmental stage may shape their interpretation of Referents items, which could explain why the Referents subscale did not meet metric invariance.

We also tested measurement invariance for Total Familism both with and without Referent subscale items. However, neither of these models demonstrated measurement invariance by parent and youth reporter. This finding was not unexpected, as pooling noninvariant with invariant items likely ‘contaminated’ the measurement model (Byrne et al., 1989; Millsap, 2012; Vandenberg & Lance, 2000). Noninvariant loadings can distort the factor structure and obscure otherwise meaningful invariance. The noninvariance of Total Familism by parent and youth reporter underscores the multifaceted nature of familism values (Choi et al., 2021; Stein et al., 2015). The various subscales may capture unique aspects of familism that are differentially interpreted by reporter (Killoren et al., 2016), thus averaging across subscales could mask important reporter‐specific variability. Together, our findings suggest that when using the Mexican American Cultural Values Scale, researchers should consider how each subscale may operate across groups/informants. Future work should formally evaluate measurement invariance across the entire Mexican American Cultural Values Scale to determine whether they can be compared across parent and youth reporters.

### Discrepancy analyses

Given that the Support and Obligations subscales demonstrated at least partial evidence of measurement invariance across reporters, we included these subscales in our discrepancy analyses. To begin, we found weak correlations (*rs* ranging from .09 to .16) among parent‐ and youth‐reported familism at youth age 11–12, corroborating previous research finding low concordance among parents and youth on familism in Hispanic/Latino/a families (Germán et al., 2009; Phinney et al., 2000; Smokowski et al., 2008). Similarly, parent‐ and youth‐reported Support and Obligations generally predicted better adolescent functioning, highlighting the importance of family values for both Hispanic/Latino/a parents and youth (Sabogal et al., 1987; Sumner et al., 2018) and corroborating the robust body of literature suggesting that familism is protective for Hispanic/Latino/a youth (Cahill et al., 2021; Rea‐Sandin, Toro, & Wilson, 2023; Stein et al., 2020; Zhao et al., 2022). However, the predictive effects of parent and youth reports tended to vary across outcomes, providing little evidence of convergent validity across reporters.

We also included the interaction between parent‐ and youth‐reported the Support and Obligations subscales, using a statistically appropriate method of testing whether reporter discrepancies at youth age 11–12 predicted early adolescent functioning 1 year later (Laird & De Los Reyes, 2013). Contrary to our hypotheses, our results suggested that discrepancies among parents and youth on familism Support and Obligations subscales do not account for unique variance over and above the main effects of each reporter. Our findings are concordant with some other work that found no evidence that discrepancies among reporters on culturally relevant measures (e.g., acculturation and familism) were associated with adolescent functioning (Lau et al., 2005; Padilla et al., 2016). However, this work contrasts with previous literature showing that greater parent–adolescent discrepancies on familism and related cultural constructs were associated with poorer adolescent functioning, although these studies used other methods (e.g., difference scores) to index reporter discrepancies (Baumann et al., 2010; Elder et al., 2005; Laird & De Los Reyes, 2013; Toro & Nieri, 2018).

### Limitations

The present study advances our understanding of the measurement characteristics of familism among parents and their early adolescents, as well as the protective role of familism values on Hispanic/Latino/an adolescent functioning. This study also comes with several limitations. First, regarding *constraints on generality*, although the ABCD Study made intentional efforts to recruit a sample that reflects the demographic diversity of the United States, the demographics of the sample are likely to be biased toward metropolitan areas and families with higher socioeconomic status (Langensee et al., 2024). Next, common method variance could have explained the significant longitudinal associations between youth‐reported familism and youth‐reported problem behaviors, school connectedness, and peer affiliation and behavior. The development and validation of other measures of youth familism, such as coded parent–child interactions, could not only mitigate potential measurement issues but further highlight youth's important contributions to the familial cultural context. For example, an objective measure (including coded parent–child interactions and experimenter ratings of children's behavior) of youth family orientation was associated with better self‐regulation in a sociodemographically diverse sample of middle childhood‐aged children (Rea‐Sandin, Li‐Grining, et al., 2023).

Another limitation relates to the developmental specificity of our study. Although the focus on familism in early adolescence addresses a gap in the literature, it is possible that some of the familism items were too abstract for youth at this age (e.g., ‘A person should always think about their family when making important decisions’), which may have resulted in differential interpretations across parent and youth reporters. Although there is evidence that latent youth familism demonstrated scalar (strong) longitudinal measurement invariance across ages 10–16 (Shi et al., 2024), the continued assessment of the ABCD study provides an opportunity to examine whether the individual subscales, particularly the referent subscale, demonstrate invariance across reporters throughout adolescence. In the present study, familism was included as an observed variable to enhance interpretability and generalizability, as well as maintain consistency with previous research. Future work should consider alternative, more sophisticated approaches, such as structural equation modeling. For example, the multi‐level latent difference score approach can test for the effect of reporter discrepancies while also accounting for measurement error (Schwartz et al., 2016). Next, the measurement invariance testing in our study was used as a preliminary step to establish that the factor structure and relative loadings were sufficiently consistent across reporters to justify examining parent–child familism discrepancies. Although we considered at least partial metric invariance as adequate for the purposes of our study, it is possible that noninvariance in intercepts could influence cross‐reporter comparisons. Future work should incorporate other approaches (e.g., alignment method) to provide additional leverage in accounting for intercept noninvariance.

Only one parent in each family reported on their endorsement of familism values in this study. Previous research has demonstrated the unique contributions of assessing familism in multiple caregivers and siblings (Knight et al., 2010, 2011; Padilla et al., 2016). Additionally, as blended families become more prevalent in the United States (Ganong & Coleman, 2018), it will be crucial to also assess how other family members' (e.g., stepparents/partners, grandparents) endorsement of familism may impact the family dynamic.

Finally, although this study was informed by acculturation gap theory, recent work has conceptualized acculturation as both bidimensional (i.e., host vs. heritage culture) and multidomain (i.e., practices, values, and identifications), suggesting that our focus on familism values represents just one facet of the broader spectrum of cultural values that contribute to youth development (Padilla, 1980; Schwartz et al., 2016). There is evidence that gaps across these different acculturation dimensions/domains also have important implications for family functioning and youth outcomes (Telzer, 2011; Unger et al., 2009), and that discrepancies in heritage culture values may be particularly salient (Schwartz et al., 2016). Thus, future research should extend beyond familism to examine how acculturation gaps in other family cultural values, such as heritage and host cultural streams, shape youth development. Nonetheless, the present study is among the largest to investigate the measurement characteristics of parent‐ and youth‐reported familism, as well as the developmental implications of familism on early adolescent well‐being in a sample of Hispanic/Latino/a youth and their parents.

### Implications

Based on measurement invariance results, associations between the Support subscale and other measures of youth functioning can be reliably compared by parent and youth reporters. Partial metric invariance was met for the Obligations subscale, suggesting that a 3‐item revised Obligation subscale should be used, rather than the full 5‐item subscale. Thus, some caution should be taken if researchers opt to include all the Obligations items when comparing parent and youth reporters. For the Referent subscale and Total Familism scale, only configural invariance could be established. This suggests that although the items follow the same loading pattern across reporters, the magnitude of loadings and the meaning of responses may differ between parents and youth. Therefore, it suggests that the comparison of mean scores cannot be made between parents and youth. It will be critical to test whether invariance by parent and youth reporter can be established in older adolescence, when youth cultural values may be more formalized as well as when adolescents have greater tangible responsibilities within the family (e.g., caretaking, cleaning, financially supporting the family; Stein et al., 2014). The Mexican American Cultural Values Scale includes other cultural constructs in addition to Familism, including Respect, Religion, and Traditional Gender Roles. It is possible that more stringent levels of measurement invariance could be established when the entire scale is considered. It is important to note that results from this study do not negate previous research comparing mean scores across parents and youth on this measure, but until more research is done, caution should be taken when comparing mean scores from full Obligation, Referent, and Total Familism in parents and their early adolescents.

## CONCLUSION

The present study highlights the important and complex role of familism values, a set of attitudes and beliefs commonly endorsed in Hispanic/Latino/a families, on adaptive adolescent functioning. Evaluating the psychometric characteristics that underlie measures of familism, as well as considering differences in the endorsement of familism among family members, strengthens our understanding of the ways in which familial cultural values emerge and manifest within families across development.

## AUTHOR CONTRIBUTIONS

**Gianna Rea‐Sandin:** Conceptualization; investigation; writing—original draft; methodology; writing—review and editing; data curation. **Sylia Wilson:** Funding acquisition; conceptualization; methodology; writing—review and editing; formal analysis; supervision.

## FUNDING INFORMATION

This research was supported by grants from the National Institute on Drug Abuse (T32DA050560 and R01DA056499) and the National Center for Advancing Translational Sciences, grant (1UM1TR004405).

## CONFLICT OF INTEREST STATEMENT

The authors have no conflicts of interest to report.

## ETHICS STATEMENT

Ethics approval statement is not applicable.

## CONSENT FOR PUBLICATION

All authors consent for the publication of this manuscript.

## PARTICIPANT CONSENT STATEMENT

In the ABCD study, all participants provided consent and assent at each wave of data collection. All consenting participants (parents of the participating youth) have also provided consent to broad data sharing, including secondary data analyses beyond the prescribed goals of the ABCD study.

## Supporting information


Appendix S1.

## Data Availability

The data that support the findings of this study are openly available in NIMH Data Archive at https://nda.nih.gov/abcd.
