# Supplementary material for: Examining parent and youth experiences of familism: Effects on youth well‐being and family dynamics
Source: J Res Adolesc. 2025 Sep 25;35(3):e70082. doi: 10.1111/jora.70082 (PMC12461440; doi:10.1111/jora.70082)
Supplement: Supplementary file 1 — Appendix S1. [file JORA-35-0-s001.docx]

Supplementary Information

Examining parent and youth experiences of familism: Effects on youth well-being and family dynamics

*Table S1.* Items Comprising the Familism Domain of Mexican American Cultural Values Scale

| Subscale | Item |
| --- | --- |
| Support | Q2. Parents should teach their children that the family always comes first |
|  | Q7. Family provides a sense of security because they will always be there for you |
|  | Q12. It is always important to be united as a family. |
|  | Q16. It is important to have close relationships with aunts/uncles, grandparents, and cousins |
|  | Q21. Holidays and celebrations are important because the whole family comes together. |
|  | Q26. It is important for family members to show their love and affection to one another. |
| Obligation | Q3. Children should be taught that it is their duty to care for their parents when their parents get old. |
|  | Q8. If a relative is having a hard time financially, one should help them out if possible. |
|  | Q13. A person should share their home with relatives if they need a place to stay. |
|  | Q17. Older kids should take care of and be role models for their younger brothers and sisters. |
|  | Q22. Parents should be willing to make great sacrifices to make sure their children have a better life. |
| Referents | Q4. Children should always do things to make their parents happy. |
|  | Q9. When it comes to important decisions, the family should ask for advice from close relatives |
|  | Q18. Children should be taught to always be good because they represent the family. |
|  | Q23. A person should always think about their family when making important decisions. |
|  | Q27. It is important to work hard and do one's best because this work reflects on the family. |

***Polynomial regression analyses for Obligations and Referent subscales and Total Familism***

**Obligations.** Parent-reported Obligations at youth age 11-12 predicted greater youth prosocial peer affiliation at youth age 12-13 (Table S5). Youth-reported Obligations predicted fewer youth-reported problem behaviors and peer victimization behaviors, as well as greater youth-reported school connectedness and prosocial peer affiliation one year later. Youth-reported Obligations had an accelerating, positive curvilinear association with parent-reported problem behaviors, and had an accelerating, negative curvilinear association with parent-reported academic performance. There were no significant interactions between parent and youth reports of Obligations at age 11-12 predicting youth functioning at age 12-13.

**Referent.** Parent-reported Referent at youth age 11-12 predicted fewer number of substances tried and greater youth-reported prosocial peer affiliation at youth age 12-13 (Table S6). Youth-reported Referent predicted fewer youth-reported problem behaviors and peer victimization behaviors, and greater youth-reported school connectedness and prosocial peer affiliation one year later. Youth-reported Referent had an accelerating, positive curvilinear association with parent-reported problem behaviors and an accelerating, negative curvilinear association with parent-reported academic performance and youth-reported prosocial peer affiliation. Although there was a statistically significant interaction between parent- and youth-reported Referent predicting youth-reported prosocial peer affiliation, it did not meet the Bonferroni adjusted alpha level of .006 and thus was not probed.

**Total Familism.** Parent-reported Total Familism at youth age 11-12 predicted fewer number of substances tried and greater youth prosocial peer affiliation at age 12-13. Youth-reported Total Familism predicted fewer youth-reported problem behaviors, and peer victimization behaviors, as well as greater prosocial peer affiliation one year later. Youth-reported Total Familism had an accelerating, positive curvilinear association with parent-reported problem behaviors and an accelerating, negative curvilinear association with parent-reported academic performance. There were no significant interactions between parent and youth reports of Total Familism at age 11-12 predicting youth functioning at age 12-13, suggesting no associations between parent-youth discrepancies in reports of Total Familism and youth functioning.

*Table S2.* Zero-Order Bivariate Correlations Between Familism and Outcome Variables

|  | Substance Use | Problem Behaviors (youth-reported) | Problem Behaviors (parent-reported) | School Connect | Academic Performance | Prosocial Peers | Rule-Break Peers | Peer Victim. | Family Conflict |
| --- | --- | --- | --- | --- | --- | --- | --- | --- | --- |
| Parent-report |  |  |  |  |  |  |  |  |  |
| Support | -.07^**^ | -.04 | -.07^**^ | .04 | .04 | .02 | .02 | -.06^*^ | -.12^***^ |
| Obligations | -.05^*^ | -.04 | -.02 | .01 | -.01 | .02 | .03 | -.02 | -.04 |
| Referent | -.09^***^ | -.07^**^ | -.05 | .04 | -.04 | .01 | .05^*^ | -.04 | -.05^*^ |
| Total | -.07^***^ | -.06^*^ | -.05 | .04 | -.01 | .02 | .04 | -.05^*^ | -.08^**^ |
| Youth-report |  |  |  |  |  |  |  |  |  |
| Support | -.05^*^ | -.14^***^ | -.10^***^ | .22^***^ | .03 | .12^***^ | -.05^*^ | -.08^***^ | -.05^*^ |
| Obligations | -.06^**^ | -.15^***^ | -.07^**^ | .19^***^ | -.03 | .10^***^ | -.04 | -.07^**^ | -.05^*^ |
| Referent | -.07^**^ | -.19^***^ | -.06^*^ | .23^***^ | -.05^*^ | .07^**^ | -.02 | -.10^***^ | -.05^*^ |
| Total | -.07^**^ | -.17^***^ | -.08^**^ | .23^***^ | -.02 | .11^***^ | -.04 | -.09^***^ | -.05^*^ |

*Note.* In this study, parent- and youth-reported Familism was assessed at the 2-year follow-up (youth age 11-12), whereas parent- and youth-reported problem behaviors, grades, school connectedness, prosocial and rule-breaking peer affiliation, aggressive peer behaviors, and family conflict were assessed at the 3-year follow-up (youth age 12-13). ^*^*p*<.050; ^**^*p*<.010; ^***^*p*<.001.

*Table S3.* Standardized Factor Loadings for Final Measurement Models of Familism by Subscale

| Subscale | Item | Parent Loading (SE) | Youth Loading (SE) |
| --- | --- | --- | --- |
| Support | Q2 | .59 (.03) | .56 (.02) |
|  | Q7 | .62 (.02) | .66 (.02) |
|  | Q12 | .70 (.02) | .77 (.02) |
|  | Q16 | .67 (.02) | .70 (.02) |
|  | Q21 | .66 (.02) | .67 (.02) |
|  | Q26 | .67 (.02) | .70 (.02) |
| Obligations | Q3 | .59 (.03) | .65 (.02) |
|  | Q8 | .53 (.03) | .67 (.02) |
|  | Q13 | .50 (.03) | .64 (.02) |
|  | Q17* | .67 (.03) | .57 (.02) |
|  | Q22* | .52 (.03) | .59 (.02) |
| Referent | Q4 | .53 (.02) | .68 (.02) |
|  | Q9 | .42 (.03) | .62 (.02) |
|  | Q18 | .85 (.01) | .79 (.01) |
|  | Q23 | .52 (.03) | .65 (.02) |
|  | Q27 | .80 (.02) | .79 (.01) |

*Note.* SE = standard error. Models were run separately by subscale. *Partial metric invariance was met for Obligations after freeing items Q17 and Q22.

*Table S4.* Standardized Factor Loadings for Final Measurement Models of Total Familism

| Item | Parent Loading (SE) | Youth Loading (SE) |
| --- | --- | --- |
| Q2 | .61 (.02) | .59 (.02) |
| Q7 | .60 (.02) | .64 (.02) |
| Q12 | .69 (.02) | .71 (.01) |
| Q16 | .64 (.02) | .70 (.02) |
| Q21 | .61 (.02) | .66 (.02) |
| Q26 | .65 (.02) | .69 (.02) |
| Q3 | .49 (.02) | .62 (.02) |
| Q8 | .60 (.02) | .67 (.02) |
| Q13 | .54 (.02) | .62 (.02) |
| Q17 | .63 (.02) | .64 (.02) |
| Q22 | .62 (.02) | .60 (.02) |
| Q4 | .43 (.02) | .63 (.02) |
| Q9 | .55 (.02) | .68 (.01) |
| Q18 | .65 (.02) | .70 (.02) |
| Q23 | .67 (.02) | .72 (.01) |
| Q27 | .64 (.02) | .71 (.01) |

*Note.* SE = standard error.

*Table S5.* Parent and youth reports of Obligations (with items Q17 and Q22) as predictors of youth functioning

| Model | Substance Use Initiation | Problem Behaviors (youth-reported) | Problem Behaviors (parent-reported) | School Connectedness | Academic Performance | Peer Affiliation – Prosocial |
| --- | --- | --- | --- | --- | --- | --- |
| Parameter | β (*SE*) | β (*SE*) | β (*SE*) | β (*SE*) | β (*SE*) | β (*SE*) |
| Intercept | **-1.507 (.20)^***^** | **-0.490 (.08)^***^** | 0.142 (.09) | -0.023 (.08) | **-0.451 (.08)^***^** | **0.176 (.08)^*^** |
| Age | **0.111 (.06)^*^** | 0.006 (.02) | -0.028 (.03) | **-0.071 (.02)^**^** | -0.023 (.02) | 0.040 (.02) |
| Sex | -0.206 (.11) | **0.322 (.05)^***^** | **-0.145 (.05)^**^** | 0.036 (.05) | **0.346 (.05)^***^** | -0.069 (.05) |
| Household Income | 0.107 (.07) | **-0.068 (.03)^*^** | -0.049 (.04) | 0.056 (.03) | **0.106 (.03)^***^** | **0.158 (.03)^***^** |
| Parent Education | **0.145 (.08)^*^** | 0.050 (.04) | 0.062 (.04) | -0.011 (.03) | **0.132 (.03)^***^** | **0.074 (.03)^*^** |
| Parent Obligations | -0.088 (.06) | -0.029 (.03) | 0.001 (.03) | 0.023 (.03) | 0.009 (.03) | **0.059 (.03)^*^** |
| Youth Oblig. | -0.068 (.06) | **-0.120 (.03)^***^** | -0.036 (.03) | **0.177 (.03)^***^** | -0.045 (.03) | **0.119 (.03)^***^** |
| Parent Obligations^2^ | - | 0.001 (.02) | 0.016 (.02) | -0.004 (.02) | -0.015 (.02) | -0.034 (.02) |
| Youth Obligations^2^ | - | 0.007 (.02) | **0.074 (.02)^***^** | 0.004 (.02) | **-0.045 (.02)^*^** | -0.012 (.02) |
| Parent X Youth Obligations | -0.103 (.06) | 0.024 (.03) | -0.015 (.03) | -0.003 (.02) | -0.007 (.02) | -0.008 (.03) |
| Model | Peer Affiliation – Rule-Break | Peer Victimization | Family Conflict |  |  |  |
| Parameter | β (*SE*) | β (*SE*) | β (*SE*) |  |  |  |
| Intercept | 0.096 (.09) | **0.188 (.08)^*^** | 0.129 (.08) |  |  |  |
| Age | **0.121 (.02)^***^** | 0.036 (.02) | 0.005 (.02) |  |  |  |
| Sex | -0.079 (.05) | **-0.121 (.05)^**^** | -0.047 (.04) |  |  |  |
| Household Income | **-0.110 (.03)^***^** | -0.029 (.03) | 0.017 (.03) |  |  |  |
| Parent Education | -0.055 (.03) | 0.059 (.03) | 0.061 (.04) |  |  |  |
| Parent Obligations | 0.001 (.03) | -0.005 (.02) | -0.005 (.03) |  |  |  |
| Youth Oblig. | -0.005 (.03) | **-0.071 (.03)^**^** | -0.020 (.02) |  |  |  |
| Parent Obligations^2^ | -0.012 (.02) | -0.018 (.02) | 0.010 (.02) |  |  |  |
| Youth Obligations^2^ | 0.032 (.02) | -0.018 (.02) | -0.003 (.01) |  |  |  |
| Parent X Youth Obligations | 0.036 (.02) | 0.008 (.02) | 0.007 (.02) |  |  |  |

*Note.* *SE* = Standard Error; ^*^*p*<.050; ^**^*p*<.010; ^***^*p*<.001. These models reflect the Obligations subscale that include items Q17 and Q22. Estimates are standardized. Substance use initiation was a binary outcome (1=yes), thus quadratic terms were not included in these regression models.

*Table S6.* Parent and youth reports of Referent as predictors of youth functioning

| Model | Substance Use Initiation | Problem Behaviors (youth-reported) | Problem Behaviors (parent-reported) | School Connectedness | Academic Performance | Peer Affiliation – Prosocial |
| --- | --- | --- | --- | --- | --- | --- |
| Parameter | β (*SE*) | β (*SE*) | β (*SE*) | β (*SE*) | β (*SE*) | β (*SE*) |
| Intercept | **-1.457 (.20)^***^** | **-0.437 (.08)^***^** | 0.173 (.09) | -0.083 (.08) | **-0.446 (.08)^***^** | **0.181 (.08)^*^** |
| Age | **0.113 (.05)^*^** | 0.002 (.02) | -0.031 (.03) | **-0.068 (.02)^**^** | -0.021 (.02) | 0.039 (.02) |
| Sex | **-0.232 (.11)^*^** | **0.299 (.05)^***^** | **-0.152 (.05)^**^** | 0.071 (.05) | **0.350 (.05)^***^** | -0.053 (.05) |
| Household Income | 0.083 (.07) | **-0.079 (.03)^*^** | -0.051 (.04) | **0.068 (.03)^*^** | **0.102 (.03)^**^** | **0.160 (.03)^***^** |
| Parent Education | 0.126 (.08) | 0.037 (.04) | 0.053 (.04) | 0.001 (.03) | **0.132 (.03)^***^** | **0.077 (.03)^*^** |
| Parent Referent | **-0.159 (.06)^**^** | -0.046 (.03) | -0.025 (.03) | 0.039 (.03) | -0.010 (.03) | **0.057 (.03)^*^** |
| Youth Referent | -0.103 (.06) | **-0.153 (.03)^***^** | -0.025 (.03) | **0.241 (.03)^***^** | -0.038 (.03) | **0.077 (.03)^**^** |
| Parent Referent^2^ | - | -0.006 (.02) | 0.009 (.02) | 0.012 (.02) | -0.010 (.02) | **-0.039 (.02)^*^** |
| Youth Referent ^2^ | - | -0.005 (.02) | **0.052 (.02)^*^** | 0.008 (.02) | **-0.062 (.02)^***^** | **-0.041 (.02) ^*^** |
| Parent X Youth Referent | -0.043 (.05) | 0.026 (.03) | 0.019 (.03) | 0.004 (.02) | 0.004 (.02) | **0.054 (.02)^*^** |
| Model | Peer Affiliation – Rule-Break | Peer Victimization | Family Conflict |  |  |  |
| Parameter | β (*SE*) | β (*SE*) | β (*SE*) |  |  |  |
| Intercept | 0.113 (.09) | **0.217 (.09)^*^** | 0.125 (.08) |  |  |  |
| Age | 0.120 (.02)**^***^** | 0.037 (.02) | 0.004 (.02) |  |  |  |
| Sex | -0.084 (.05) | **-0.139 (.05)^**^** | -0.048 (.04) |  |  |  |
| Household Income | -0.108 (.03)**^***^** | -0.024 (.03) | 0.020 (.03) |  |  |  |
| Parent Education | -0.056 (.03) | 0.040 (.03) | 0.060 (.04) |  |  |  |
| Parent Referent | 0.017 (.03) | -0.011 (.03) | 0.006 (.03) |  |  |  |
| Youth Referent | -0.024 (.03) | **-0.123 (.03)^***^** | -0.014 (.02) |  |  |  |
| Parent Referent^2^ | -0.018 (.02) | -0.009 (.02) | 0.025 (.02) |  |  |  |
| Youth Referent ^2^ | 0.023 (.02) | -0.032 (.02) | -0.013 (.01) |  |  |  |
| Parent X Youth Referent | 0.047 (.02) | 0.046 (.02) | 0.004 (.02) |  |  |  |

*Note.* *SE* = Standard Error; ^*^*p*<.050; ^**^*p*<.010; ^***^*p*<.001. Estimates are standardized. Substance use initiation was a binary outcome (1=yes), thus quadratic terms were not included in these regression models.

*Table S7.* Parent and youth reports of Total Familism as predictors of youth functioning

| Model | Substance Use Initiation | Problem Behaviors (youth-reported) | Problem Behaviors (parent-reported) | School Connectedness | Academic Performance | Peer Affiliation – Prosocial |
| --- | --- | --- | --- | --- | --- | --- |
| Parameter | β (*SE*) | β (*SE*) | β (*SE*) | β (*SE*) | β (*SE*) | β (*SE*) |
| Intercept | **-1.480 (.20)^***^** | **-0.452 (.08)^***^** | 0.171 (.09) | -0.036 (.08) | **-0.461 (.08)^***^** | **0.172 (.08)^***^** |
| Age | **0.114 (.05)^*^** | 0.002 (.02) | -0.032 (.03) | **-0.069 (.02)^**^** | -0.021 (.02) | 0.042 (.02) |
| Sex | **-0.219 (.11)^*^** | **0.320 (.05)^***^** | **-0.146 (.05)^**^** | 0.040 (.05) | **0.348 (.05)^***^** | -0.067 (.05) |
| Household Income | 0.101 (.07) | **-0.076 (.03)^*^** | -0.057 (.04) | **0.066 (.03)^*^** | **0.107 (.03)^***^** | **0.160 (.03)^***^** |
| Parent Education | 0.131 (.08) | 0.046 (.04) | 0.060 (.04) | -0.010 (.03) | **0.133 (.03)^***^** | **0.074 (.03)^*^** |
| Parent Familism | **-0.150 (.06)^*^** | -0.042 (.03) | -0.034 (.03) | 0.036 (.03) | 0.013 (.03) | **0.060 (.03)^*^** |
| Youth Familism | -0.076 (.06) | **-0.156 (.03)^***^** | -0.045 (.03) | **0.228 (.03)^***^** | -0.036 (.03) | **0.116 (.03)^***^** |
| Parent Familism^2^ | - | 0.001 (.02) | -0.006 (.02) | 0.001 (.03) | -0.007 (.02) | -0.026 (.02) |
| Youth Familism^2^ | - | -0.027 (.02) | **0.062 (.02)^**^** | 0.016 (.02) | **-0.047 (.02)^**^** | -0.015 (.02) |
| Parent X Youth Familism | -0.065 (.05) | 0.025 (.02) | 0.009 (.03) | -0.003 (.02) | -0.001 (.02) | 0.022 (.02) |
| Model | Peer Affiliation – Rule-Break | Peer Victimization | Family Conflict |  |  |  |
| Parameter | β (*SE*) | β (*SE*) | β (*SE*) |  |  |  |
| Intercept | 0.092 (.08) | **0.200 (.09)^*^** | 0.134 (.07) |  |  |  |
| Age | **0.119 (.02)^***^** | 0.042 (.02) | 0.003 (.02) |  |  |  |
| Sex | -0.078 (.05) | **-0.121 (.05)^**^** | -0.051 (.04) |  |  |  |
| Household Income | **-0.116 (.03)^***^** | -0.032 (.03) | 0.015 (.03) |  |  |  |
| Parent Education | -0.050 (.03) | 0.049 (.03) | 0.057 (.03) |  |  |  |
| Parent Familism | 0.006 (.03) | -0.033 (.03) | -0.023 (.03) |  |  |  |
| Youth Familism | -0.018 (.03) | **-0.106 (.03)^***^** | -0.022 (.02) |  |  |  |
| Parent Familism^2^ | -0.012 (.02) | -0.014 (.02) | 0.010 (.01) |  |  |  |
| Youth Familism^2^ | 0.034 (.02) | -0.032 (.02) | -0.009 (.01) |  |  |  |
| Parent X Youth Familism | 0.020 (.02) | 0.024 (.02) | 0.002 (.02) |  |  |  |

*Note.* SE=Standard Error; ^*^*p*<.050; ^**^*p*<.010; ^***^*p*<.001. Estimates are standardized. Substance use initiation was a binary outcome (1=yes), thus quadratic terms were not included in these regression models.

*Table S8.* Parent and youth reports of Support as predictors of youth functioning (multiple imputation results)

| Model | Substance Use Initiation | Problem Behaviors (youth-reported) | Problem Behaviors (parent-reported) | School Connectedness | Academic Performance | Peer Affiliation – Prosocial |
| --- | --- | --- | --- | --- | --- | --- |
| Parameter | β (*SE*) | β (*SE*) | β (*SE*) | β (*SE*) | β (*SE*) | β (*SE*) |
| Intercept | **-1.516 (.20)^***^** | **-0.473 (.08)^***^** | **0.190 (.09)^*^** | -0.005 (.08) | **-0.490 (.08)^***^** | 0.151 (.08) |
| Age | **0.113 (.06)^*^** | 0.002 (.03) | -0.037 (.03) | **-0.066 (.02)^**^** | -0.018 (.02) | 0.043 (.02) |
| Sex | -0.201 (.11) | **0.335 (.05)^***^** | **-0.134 (.06)^*^** | 0.017 (.05) | **0.347 (.05)^***^** | -0.081 (.05) |
| Household Income | 0.097 (.07) | -0.063 (.03) | -0.060 (.04) | 0.053 (.03) | **0.112 (.03)^***^** | **0.148 (.03)^***^** |
| Parent Education | 0.151 (.08) | 0.051 (.04) | 0.061 (.04) | -0.019 (.03) | **0.136 (.03)^***^** | **0.073 (.03)^*^** |
| Parent Support | **-0.126 (.06)^*^** | -0.016 (.03) | -0.065 (.03) | 0.026 (.03) | 0.042 (.03) | 0.032 (.03) |
| Youth Support | -0.032 (.06) | **-0.149 (.03)^***^** | **-0.066 (.03)^*^** | **0.211 (.03)^***^** | -0.020 (.03) | **0.145 (.03)^***^** |
| Parent Support^2^ |  | 0.015 (.02) | -0.020 (.02) | -0.008 (.02) | 0.006 (.02) | -0.021 (.02) |
| Youth Support^2^ |  | **-0.041 (.02)^*^** | 0.037 (.02) | 0.018 (.02) | -0.028 (.02) | 0.019 (.02) |
| Par. X Youth Support | 0.004 (.05) | 0.011 (.03) | 0.011 (.03) | 0.009 (.02) | -0.003 (.02) | 0.018 (.02) |
| Model | Peer Affiliation – Rule-Break | Peer Victimization | Family Conflict |  |  |  |
| Parameter | β (*SE*) | β (*SE*) | β (*SE*) |  |  |  |
| Intercept | 0.080 (.08) | **0.165 (.08)^*^** | 0.090 (.07) |  |  |  |
| Age | **0.116 (.02)^***^** | 0.038 (.02) | -0.002 (.02) |  |  |  |
| Sex | -0.076 (.05) | **-0.108 (.05)^*^** | -0.047 (.04) |  |  |  |
| Household Income | **-0.108 (.03)^**^** | -0.032 (.03) | -0.002 (.03) |  |  |  |
| Parent Education | -0.052 (.03) | 0.060 (.03) | **0.074 (.04)^*^** |  |  |  |
| Parent Support | 0.002 (.03) | -0.048 (.03) | **-0.086 (.03)^**^** |  |  |  |
| Youth Support | -0.013 (.03) | **-0.097 (.03)^***^** | -0.041 (.02) |  |  |  |
| Parent Support^2^ | 0.001 (.02) | -0.016 (.02) | -0.006 (.01) |  |  |  |
| Youth Support^2^ | **0.035 (.02)^*^** | -0.018 (.02) | -0.019 (.01) |  |  |  |
| Par. X Youth Support | -0.014 (.02) | 0.022 (.02) | -0.002 (.02) |  |  |  |

*Note.* *SE* = Standard Error; ^*^*p*<.050; ^**^*p*<.010; ^***^*p*<.001. Estimates are standardized. Substance use initiation was a binary outcome (1=yes), thus quadratic terms were not included in these regression models. All models were estimated using multiple imputation (m=20).

*Table S9.* Parent and youth reports of Obligations as predictors of youth functioning (multiple imputation results)

| Model | Substance Use Initiation | Problem Behaviors (youth-reported) | Problem Behaviors (parent-reported) | School Connectedness | Academic Performance | Peer Affiliation – Prosocial |
| --- | --- | --- | --- | --- | --- | --- |
| Parameter | β (*SE*) | β (*SE*) | β (*SE*) | β (*SE*) | β (*SE*) | β (*SE*) |
| Intercept | **-1.505 (.20)^***^** | **-0.495 (.08)^***^** | 0.146 (.10) | -0.026 (.08) | **-0.448 (.08)^***^** | **0.171 (.09)^*^** |
| Age | 0.107 (.06) | 0.008 (.03) | -0.030 (.03) | **-0.070 (.02)^**^** | -0.026 (.02) | 0.035 (.02) |
| Sex | -0.214 (.11) | **0.320 (.05)^***^** | **-0.140 (.06)^*^** | 0.034 (.05) | **0.343 (.05)^***^** | -0.075 (.05) |
| Household Income | 0.103 (.08) | **-0.072 (.03)** | -0.049 (.04) | 0.060 (.03) | **0.106 (.03)^**^** | **0.155 (.03)^***^** |
| Parent Education | 0.153 (.08) | 0.054 (.04) | 0.062 (.04) | -0.016 (.03) | **0.131 (.03)^***^** | **0.074 (.03)^*^** |
| Parent Obligations | -0.043 (.06) | -0.031 (.03) | -0.008 (.03) | 0.024 (.03) | 0.019 (.03) | **0.058 (.03)^*^** |
| Youth Obligations | -0.069 (.06) | **-0.113 (.03)^***^** | -0.029 (.03) | **0.164 (.03)^***^** | **-0.065 (.03)^*^** | **0.094 (.03)^***^** |
| Parent Obligations^2^ |  | -0.008 (.02) | 0.028 (.02) | -0.004 (.02) | -0.008 (.02) | -0.021 (.02) |
| Youth Obligations^2^ |  | 0.024 (.02) | **0.049 (.02)^*^** | 0.008 (.02) | **-0.052 (.02)^**^** | -0.009 (.02) |
| Par. X Youth Obligations | -0.066 (.06) | 0.027 (.03) | -0.027 (.03) | 0.001 (.02) | -0.002 (.02) | -0.023 (.03) |
| Model | Peer Affiliation – Rule-Break | Peer Victimization | Family Conflict |  |  |  |
| Parameter | β (*SE*) | β (*SE*) | β (*SE*) |  |  |  |
| Intercept | 0.071 (.09) | 0.151 (.08) | 0.120 (.07) |  |  |  |
| Age | **0.121 (.02)^***^** | 0.037 (.02) | 0.003 (.02) |  |  |  |
| Sex | -0.080 (.05) | **-0.118 (.05)^*^** | -0.053 (.04) |  |  |  |
| Household Income | **-0.112 (.03)^***^** | -0.029 (.03) | 0.002 (.03) |  |  |  |
| Parent Education | -0.055 (.03) | 0.061 (.03) | **0.071 (.04)^*^** |  |  |  |
| Parent Obligations | 0.003 (.03) | -0.001 (.02) | -0.020 (.03) |  |  |  |
| Youth Obligations | -0.029 (.03) | **-0.071 (.03)^**^** | -0.016 (.02) |  |  |  |
| Parent Obligations^2^ | 0.011 (.03) | -0.005 (.02) | 0.027 (.01) |  |  |  |
| Youth Obligations^2^ | **0.096 (.03)^**^** | -0.004 (.02) | 0.001 (.01) |  |  |  |
| Par. X Youth Obligations | 0.015 (.04) | -0.011 (.02) | 0.005 (.02) |  |  |  |

*Note.* *SE* = Standard Error; ^a^=parent-reported; ^b^=youth-reported. ^*^*p*<.050; ^**^*p*<.010; ^***^*p*<.001. These models reflect the Obligations subscale without items Q17 and Q22. Substance use initiation was a binary outcome (1=yes), thus quadratic terms were not included in these regression models. All models were estimated using multiple imputation (m=20).
